# Predictive and prognostic value of total tumor load in sentinel lymph nodes in breast cancer patients after neoadjuvant treatment using one-step nucleic acid amplification: the NEOVATTL study

**DOI:** 10.1007/s12094-020-02530-4

**Published:** 2021-01-31

**Authors:** B. Vieites, M. Á. López-García, M. D. Martín-Salvago, C.L. Ramirez-Tortosa, R. Rezola, M. Sancho, L. López-Vilaró, F. Villardell, O. Burgués, B. Fernández-Rodriguez, L. Alfaro, V. Peg

**Affiliations:** 1 Department of Pathology, Hospital Universitario Virgen del Rocío, Sevilla, Spain; 2 Department of Pathology, Hospital Universitario Materno-Infantil, Jaén, Spain; 3 Department of Pathology, Onkologikoa Kutxa Fundazioa, Donostia, Spain; 4 Department of Pathology, Hospital Universitario de Salamanca, Salamanca, Spain; 5 Department of Pathology, Hospital de La Santa Creu I Sant Pau, Barcelona, Spain; 6 Hospital Universitario Arnau de Vilanova, Lérida, Spain; 7 Department of Pathology, Hospital Clínico Universitario de Valencia, Valencia, Spain; 8 Department of Pathology, Complejo Hospitalario, Universitario de Santiago, Santiago de Compostela, Spain; 9 Department of Gynaecology and Obstetrics, Hospital Universitario Virgen del Rocío, Sevilla, Spain; 10 Department of Pathology, Hospital Universitari Vall d’Hebron, Barcelona, Spain; 11 CIBERONC (Centro de Investigación Biomédica en Red de Cáncer), Instituto de Salud Carlos III, Madrid, Spain

**Keywords:** Breast cancer, Disease-free survival, Neoadjuvant systemic therapy, OSNA, Sentinel lymph node, Total tumor load

## Abstract

**Objective:**

To evaluate the predictive and prognostic value of total tumor load (TTL) in sentinel lymph nodes (SLNs) in patients with infiltrating breast cancer after neoadjuvant systemic therapy (NST).

**Methods:**

This retrospective multicenter study used data from a Spanish Sentinel Lymph Node database. Patients underwent intraoperative SLN biopsy after NST. TTL was determined from whole nodes using a one-step nucleic acid amplification (OSNA) assay and defined as the total sum of CK19 mRNA copies in all positive SLNs. Cox-regression models identified independent predictive variables, which were incorporated into a nomogram to predict axillary non-SLN metastasis, and identified prognostic variables for incorporation into a disease-free survival (DFS) prognostic score.

**Results:**

A total of 314 patients were included; most had no lymph node involvement prior to NST (cN0; 75.0% of patients). Most received chemotherapy with or without biologic therapy (91.7%), and 81 patients had a pathologic complete response. TTL was predictive of non-SLN involvement (area under the concentration curve = 0.87), and at a cut-off of 15,000 copies/µL had a negative predictive value of 90.5%. Nomogram parameters included log (TTL + 1), maximum tumor diameter and study-defined NST response. TTL was prognostic of disease recurrence and DFS at a cut-off of 25,000 copies/µL. After a 5-year follow-up, DFS was higher in patients with ≤ 25,000 copies/µL than those with > 25,000 (89.9% vs. 70.0%; *p* = 0.0017).

**Conclusions:**

TTL > 15,000 mRNA copies/µL was predictive of non-SLN involvement and TTL > 25,000 mRNA copies/µL was associated with a higher risk of disease recurrence in breast cancer patients who had received NST.

**Supplementary Information:**

The online version contains supplementary material available at 10.1007/s12094-020-02530-4.

## Introduction

In 2018, breast cancer was the most common cancer in women worldwide, with 2.1 million incident cases, representing 25.4% of all cancers in women [1]. With the drive for breast-conserving surgery, as well as early treatment for presumed micrometastatic disease, there is an increasing number of early-stage breast cancer patients treated with neoadjuvant systemic therapy (NST) prior to surgery [2, 3]. Assessment of regional lymph node status provides important prognostic information, and in the case of patients who have undergone NST, sentinel lymph node biopsy (SLNB) has gained attention because of its potential for less invasive management of the axilla; it should ideally take place after, not prior to, NST [4]. One-step nucleic acid amplification (OSNA) assay is an accurate and reliable option for intraoperative molecular analysis of SLN status [5].

The OSNA assay (Sysmex, Kobe, Japan) analyzes the whole SLN through a semi-quantitative result based on the detection and real-time reverse transcription-loop-mediated isothermal amplification (RT-LAMP) of cytokeratin 19 (CK19) mRNA, and provides accurate intraoperative detection of lymph node metastases [6–11]. High concordance between OSNA and conventional techniques in the detection of breast cancer cells has been observed [6, 12]. OSNA is a commonly used axillary staging method in Europe and Asia, but there has been debate on its use in patients who have undergone NST [5]. There is, however, evidence that CK19 expression is preserved in breast cancer cells after NST [12, 13] and some studies have shown identification of SLN metastases in patients following NST is similar to that in patients who have not undergone NST [14, 15].

In addition, the role of total tumor load (TTL; amount of CK19 mRNA copies in all positive SLN) for assessing non-SLN involvement and patient prognosis continues to be an area of active investigation in early breast cancer [16, 17], with a continued focus on sparing patients from axillary lymph node dissection (ALND). However, data on the predictive and prognostic value of TTL derived from intra-operative OSNA assay in breast cancer patients after NST are lacking. The aim of this retrospective study was to evaluate the predictive and prognostic value of TTL determined from OSNA assessment of SLNs in the neoadjuvant setting in breast cancer patients.

## Methods

### Study population

A historical cohort of patients diagnosed with infiltrating breast cancer between 2009 and 2012 was selected for this study. Patients where included if they had received NST and had undergone SLNB and evaluation of all complete excised SLNs by OSNA. Patients were excluded from this analysis if they were diagnosed with carcinoma in situ without an infiltrating component or any other concomitant neoplasia.

Due to the retrospective nature of this study, selection of patients for SLNB was per usual institutional practice, i.e. patients with minimal or null node involvement evaluated by imaging after NST and deemed to be candidates who would benefit from avoiding axillary dissection. Likewise, NST was administered according to the treating oncologists’ decision and per institutional protocols.

### Data and data source

Patient data were from eight Spanish hospitals on the LYNOLOG database, an online database created by the Spanish Society for the Study of Sentinel Node (*Sociedad Española para el Estudio del Ganglio Centinela*, SEEGC). The Spanish Society of Senology and Breast Disease (*Sociedad Española de Senología y Patología Mamaria*, SESPM) gave permission to use this database and the Spanish Society of Anatomy and Pathology (*Sociedad Española de Anatomía Patológica,* SEAP) acted as the guarantor. The study was conducted in accordance with the Declaration of Helsinki and guidelines for Good Clinical Practice, after obtaining PEIBA Ethics Committee (*Portal de Ética de la Investigación Biomédica de Andalucía*) approval.

Clinical, pathologic and treatment data were collected, including patient characteristics (sex), NST (type, start and end date), radiotherapy received (yes/no, location [breast ± axilla]), tumor characteristics (histologic subtype and grade, presence of in situ carcinoma, lymphovascular invasion, hormone receptor status, human epidermal growth factor receptor 2 [HER2] status, Ki67 proliferation index score, TNM stage before and after NST [cTNM and ycTNM, respectively]), and histologic tumor response according to the Miller–Payne grading system [18] (see Supplementary Methods in Online Resource 1 for a complete list).

### Pre-operative sentinel node assessment

Axillary lymph nodes (ALNs) were clinically evaluated per institutional protocol, i.e. usually by clinical and ultrasound examination. Any clinically suspicious ALN underwent histologic confirmation by fine-needle aspiration or core biopsy. SLNB was performed if ≤ 1 ALN was positive for metastasis; in general, ALND was performed if some ALNs were positive (with no SLN analysis undertaken).

### Detection and OSNA assay of SLNs

Each institution’s pathology department examined each whole lymph node using the OSNA assay, composed of the automated Gene Amplification Detector RD-100i and the LYNOAMP BC gene amplification reagent (Sysmex Corporation, Kobe, Japan). The assay technique has been described in detail previously [10]. Briefly, the SLN was homogenized in 4 mL of LYNORHAG lysis buffer (Sysmex Corporation, Kobe, Japan). A 2 µL aliquot was used for automated real-time amplification of CK19 mRNA with the ready-to-use LYNOAMP reagent on the RD-100i analyzer. Lymph node metastatic status was determined according to the manufacturer’s criteria: metastatic (OSNA positive) ≥ 2.5 × 10^2^ CK19 mRNA copies/µL (hereafter referred to as copies/µL), and non-metastatic (OSNA negative) < 2.5 × 10^2^ copies/µL [10].

### Study definitions

TTL was defined as the total sum of CK19 mRNA copies in all positive SLN, and TTL cut-offs defined per Peg et al. [19]. In this analysis, pathologic complete response to NST was defined as a lack of invasive disease according to the TNM approach in the AJCC Cancer Staging Manual (8th ed) [20], i.e. as no invasive breast tumor (by histopathology) and no metastases in the nodes (by OSNA assay of SLN or histologic analysis of non-SLNs). Treatment response was evaluated histologically using the Miller–Payne grading system. Disease-free survival (DFS) was defined as the period of time with no evidence of disease.

### Study objective

The primary objectives were to determine the predictive value of TTL for the diagnosis of non-SLN metastases in patients after NST and to determine the prognostic value of TTL in determining 5-year DFS.

### Statistics

Descriptive statistics were used for quantitative variables (mean, standard deviation [SD], median, interquartile range [IQR, P25–P75], minimum and maximum) and categorical variables (absolute or relative frequency with corresponding 95% confidence interval [CI]).

The sensitivity, specificity, positive and negative predictive values, accuracy and error rate of TTL was calculated. Area under the receiver operating characteristic (ROC) curve (AUC) was determined to evaluate the predictive value of TTL for axillary non-SLN involvement.

Cox-regression models were constructed to assess the main study objectives. Univariate and multivariate analyses were conducted to identify factors predictive of non-SLN involvement, from which a nomogram was constructed to predict non-SLN involvement. For assessment of prognostic factors, an initial univariate analysis included time to event (recurrence) as the dependent variable, and all potential prognostic factors, including TTL, as independent variables. Thereafter, independent variables reaching a *p*-value < 0.25 were included in the multivariate analysis. DFS was calculated by Kaplan–Meier survival analysis. A score to estimate 5-year DFS was also calculated (Supplementary Methods—Online Resource 1).

The planned sample size was 296 patients, determined using estimates from previous studies (disease recurrence rate of 16% [21] and SD of 1.3 [22]), and calculated using PASS software version 13 (NCSS Statistical Software). This would provide a statistical power of 90% with an alpha-error of 5%. Statistical analyses were performed using SAS software version 9.4 (SAS Institute Inc., Cary, NC, USA).

## Results

### Patients and treatment

The study cohort consisted of 314 patients, of whom only one was male (Table 1). Prior to NST, most patients did not have lymph node involvement (cN0; 75.0% of patients assessed), 24.0% were classified as cN1 and 1.0% as cN2 (Table 2). Of the 231 cN0 patients, 129 underwent ALN histologic examination prior to NST, of whom 81 (25.8%) had no metastatic nodal involvement and 48 (15.3%) had metastatic ALN (but most with signs of remission *after* NST); 3 of these 48 patients underwent a SLNB both before and after NST.

**Table 1:** Patient demographic and clinical characteristics

Characteristic	All included patients (*n* = 314)
At diagnosis	
Gender	
Male, *n* (%)	1 (0.3)
Female, *n* (%)	313 (99.7)
Tumor type, *n* (%)	
Ductal	262 (84.0)
Other	50 (16.0)
Neoadjuvant systemic therapy	
Duration, mean ± SD (months)	4.8 ± 2.2
Number of cycles, mean ± SD	8.7 ± 3.3
Type, *n* (%)	
Chemotherapy ± biologic therapy	288 (91.7)
Chemotherapy + hormonal	6 (1.9)
Hormonal therapy ± biologic therapy	20 (6.4)
Adjuvant radiotherapy, *n* (%)	
Breast ± axillary nodes	244 (77.7)
Breast + axillary nodes	50 (15.9)
Breast + axillary nodes + ALND	31 (9.9)

*ALND* axillary lymph node dissection, *SD* standard deviation

**Table 2:** Pathologic tumor characteristics before and after neoadjuvant systemic therapy (NST)

Maximum tumor size	Before NST *n* = 267	After NST
		*n* = 309
≤ 20 mm	25 (9.4)	125 (40.5)
> 20 mm	242 (90.6)	103 (33.3)
Histologic subtype	*n* = 312	*n* = 312
Ductal (including NOS)	262 (84.0)	182 (58.3)
Others	50 (16.0)	49 (15.7)
Histologic grade	*n* = 296	*n* = 307
G1	36 (12.2)	62 (20.2)
G2 and G3	260 (87.8)	164 (53.4)
In situ carcinoma	*n* = 314	*n* = 261
No	250 (79.6)	162 (62.1)
Yes	64 (20.4)	99 (37.9)
Lymphovascular infiltration	*n* = 314	*n* = 286
No	199 (63.4)	192 (67.1)
Yes	115 (36.6)	94 (32.9)
ER	*n* = 311	*n* = 111
Negative	84 (27.0)	21 (18.9)
Positive (≥ 1%)	227 (73.0)	90 (81.1)
PR	*n* = 305	*n* = 111
Negative	116 (38.0)	55 (49.5)
Positive (≥ 1%)	189 (62.0)	56 (50.5)
HER2	*n* = 314	*n* = 293
Negative	233 (74.2)	278 (94.9)
Positive (≥ 1%)	81 (25.8)	15 (5.1)
Ki67	*n* = 310	*n* = 107
> 20%	184 (59.4)	35 (32.7)
≤ 20%	126 (40.6)	72 (67.3)
TNM stage	cTNM	ycTNM
0.T	*n* = 314	*n* = 309
In situ	NA	5 (1.6)
0	NA	77 (24.9)
1	26 (8.3)	115 (37.2)
2	228 (72.6)	94 (30.4)
3	52 (16.6)	17 (5.5)
4	8 (2.5)	1 (0.3)
N	*n* = 308	*n* = 314
0	231 (75.0)	194 (61.8)
1	74 (24.0)	95 (30.3)
2	3 (1.0)	24 (7.6)
3	–	1 (0.3)
M	*n* = 312	*n* = 314
0	312 (100.0%)	314 (100.0)

All data are *n* (%) unless otherwise specified

*G* grade, *TTL* total tumor load, *pCR* pathologic complete response, *ER* estrogen receptor, *HER2* human epidermal growth factor receptor-2, *PR* progesterone receptor, *cTNM* clinical TNM tumor stage, *ycTNM* TNM stage after neoadjuvant therapy

Neoadjuvant ST for most patients was chemotherapy with or without biologic or anti-human epidermal growth factor receptor-2 (HER2) agents (91.7%), whereas only 6.4% of patients received hormone therapy (with or without biologic/HER2 treatment) (Table 1). Adjuvant breast irradiation was administered to the majority of patients (*n* = 244), of whom 50 also received ALN irradiation. Thirty-one patients underwent both ALND and axillary irradiation; of these, 15 had positive SLNs, but no further axillary treatment was performed. The maximum TTL for these cases was 6,700 copies/µL.

### Histopathologic outcomes

After NST, 69 of 309 patients (22.3%) had a pCR. More patients had a maximum tumor size of ≤ 20 mm and better histologic differentiation and staging after NST than before, and more patients were ycN1 after NST than those who were designated cN1 prior to NST (30.3% vs. 24.0%; Table 2). Pathologic tumor features pre- versus post-NST by TTL are shown in Table S1 in Online Resource 2.

During surgery (after NST), OSNA was performed in all patients for molecular analysis of SLNs, and ALND added in 127 (40.4%) for whom OSNA revealed positive SLNs. Pathologic and molecular analysis results are shown in Table 3. A total of 196 patients (62.4%) were considered to have negative results for residual cancer burden in axillary nodes (TTL < 250 mRNA copies/µL). During surgery, one or two SLN were removed in 227 (72.3%) patients whereas 87 patients (27.7%) had more than two removed (Table 3).

**Table 3:** Clinicopathologic characteristics of patients derived from histologic and molecular analyses

Variables	
Total tumor load, copies/µL	*n* = 314
Mean ± SD	42,314 ± 248,208
95% CI	14,754–69,874
Median (min–max)	0 (0–3,400,000)
Time since diagnosis, months	*n* = 314
Mean ± SD	6.7 ± 1.6
95% CI	6.5–6.8
Median (min–max)	6.6 (0.0–11.8)
No. of SLN excised^a^, *n* (%)	*n* = 314
1 or 2	227 (72.3)
> 2	87 (27.7)
No. of metastatic SLN^a^, *n* (%)	*n* = 314
0	196 (62.4)
1–3	111 (35.4)
> 3	7 (2.2)
ALND, *n* (%)	*n* = 314
No	187 (59.6)
Yes	127 (40.4)
No. of non-SLN excised, *n* (%)	*n* = 314
< 9	219 (69.7)
≥ 9	95 (30.3)
No. of metastatic non-SLN, *n* (%)	*n* = *314*
0	268 (85.4)
1–3	23 (7.3)
> 3	23 (7.3)
Tumor pathologic response, *n* (%)	*n* = 301
Grade 1	22 (7.3)
Grade 2	42 (14.0)
Grade 3	114 (37.9)
Grade 4	38 (12.6)
Grade 5	85 (28.2)

^a^At the time of the SLNB after NST

*ALND* axillary lymph node dissection, *CI* confidence interval, *no*. number, *SD* standard deviation, *SLN* sentinel lymph node

### Patient outcomes by TTL

Median follow-up after surgery was 5.2 (IQR 1.5) years. A total of 17 (5.4%) patients died during follow up, of whom 7 (41.2%) had a TTL of > 250 copies/µL; 5 (29.4%) had a TTL of 250–25,000 copies/µL, and 5 (29.4%) had a TTL of > 25,000 copies/µL. Distant metastases were detected in 35 patients (11.1%), of whom 16 (45.7%), 9 (25.7%) and 10 (28.6%) were in each TTL category, respectively. Respective disease recurrence rates were 51.3%, 23.1%, and 25.6%, with an overall disease recurrence rate of 12.4% (39 patients).

### Predictive value of TTL and clinicopathologic factors for non-SLN involvement

The ROC curve analysis demonstrated univariate concordance between TTL and non-SLN involvement (Online Resource 3, Fig. S1), with an AUC of 0.87 (95% CI 0.823–0.918). When the TTL-cutoff was fixed at 250 copies/µL, 5,000 copies/µL and 15,000 copies/µL, respective values for sensitivity were 95.7%, 63.0% and 43.5%, and for specificity were 72.4%, 86.2% and 92.9%, positive predictive values were 37.3%, 43.9% and 51.3%, and negative predictive values were 99.0%, 93.1% and 90.5%.

**Fig. 1:**
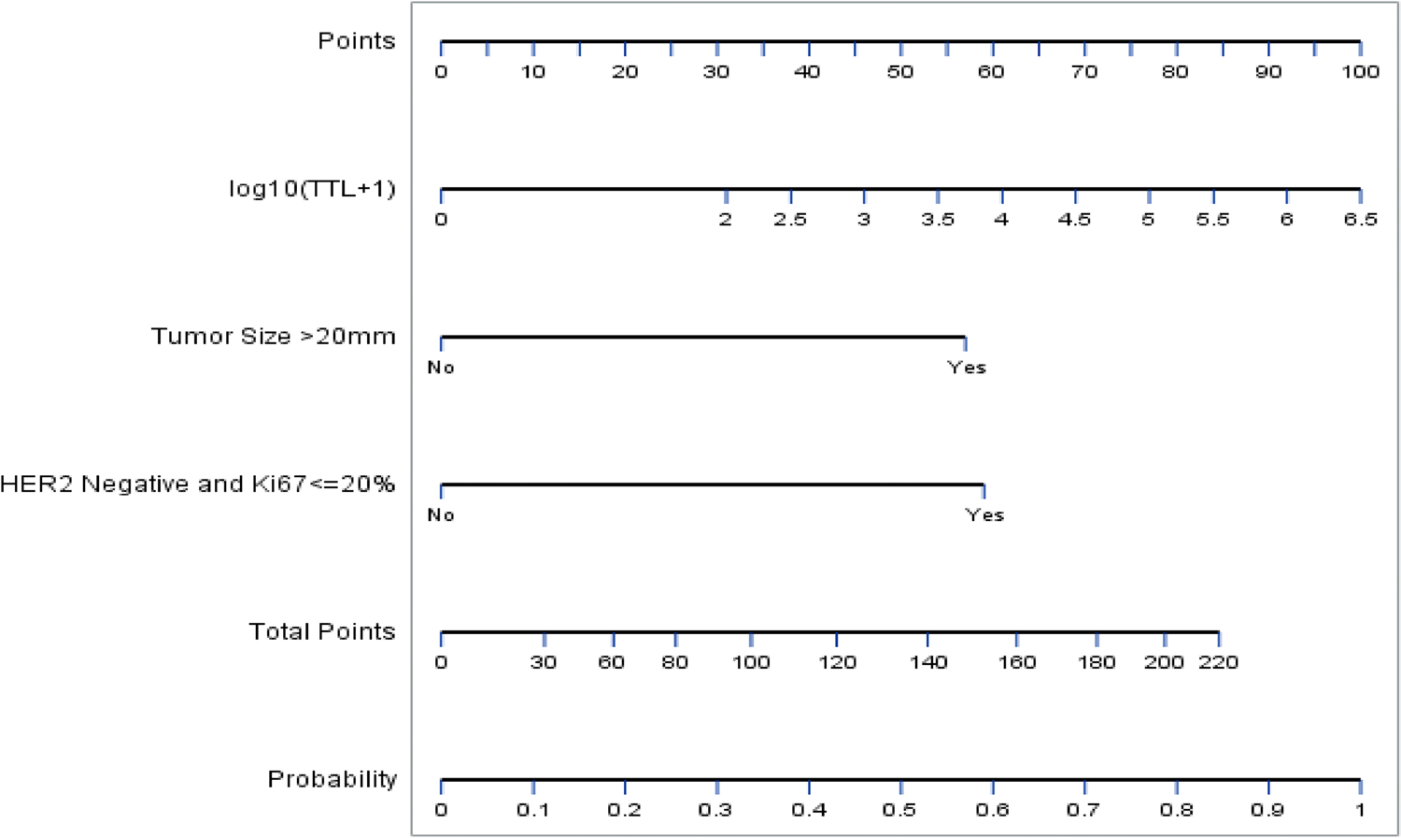
Nomogram to predict axillary non-SLN involvement in breast cancer patients after neoadjuvant systemic therapy. Probability refers to the probability of the presence of non-SNL metastasis. HER2, human epidermal growth factor receptor-2; Ki67, proliferation index; SLN, sentinel lymph node, TTL, total tumor load

Univariate and multivariate logistic regression analyses were performed to investigate the factors predictive of non-SLN metastasis (Table 4). Independent predictive factors significantly associated with non-SLN metastasis were: TTL (log_10_(TTL + 1)), treatment response (defined as patients with HER2-negative status and Ki67 ≤ 20% from needle biopsy), and maximum tumor diameter (*p* = 0.0206, 0.0069 and 0.0080, respectively). The nomogram for prediction of non-SLN status is shown in Fig. 1.

**Table 4:** Summary of univariate and multivariate analysis for non-SLN metastasis

Variable		Univariate analysis		Multivariate analysis	
		OR (95% CI)	p value	OR (95% CI)	p value
Clinical	Max. tumor diameter				
	> 20 mm vs. ≤ 20 mm	3.23 (1.52–6.86)	0.0023	3.12 (1.34–7.22)	0.0080
	Tumor grade				
	G1 vs. pCR	13.81 (1.62–117.94)	0.0323		
	G2/ G3 vs. pCR	11.97 (1.51–94.69)	0.0494		
	LVI	2.47 (1.15–5.27)	0.0197		
	Yes vs. No				
Pathologic	Log_10_(TTL + 1)	1.54 (1.20–1.96)	0.0006	1.36 (1.05–1.75)	0.0206
	TTL				
	> 25,000 vs. ≤ 25,000 copies/µL	3.37 (1.48–7.66)	0.0038		
	HER2 status				
	Positive vs. Negative	0.26 (0.08–0.80)	0.0186		
	Ki67				
	> 20% vs. ≤ 20%	0.31 (0.14–0.66)	0.0025		
Treatment response	HER2-negative and Ki67 ≤ 20% from needle biopsy			3.22 (1.38–7.54)	0.0069
	Yes vs. No	3.58 (1.68–7.66)	0.0010		
	Miller–Payne response^a^	0.69 (0.48–0.98)	0.0382		

^a^Comparison among grades

*CI* confidence interval, *HER2* human epidermal growth factor receptor 2, *LVI* lymphovascular infiltration, *OR* odds ratio, *max*. maximum, *pCR* pathologic complete response, *SLN* sentinel lymph node, *TTL* total tumor load

### Prognostic factors for recurrence and DFS

Variables reaching significance of *p*<0.25 in a univariate analysis of factors prognostic of disease recurrence were included in a multivariate analysis (data not shown). In the multivariate analysis, independent predictors of disease recurrence were TTL, Ki67 before NST, Miller–Payne grading, and cN status. A TTL of >25,000 copies/μL was associated with a threefold risk of recurrence compared with a TTL of ≤ 25,000 copies/μL (hazard ratio [HR] 2.95; 95% CI 1.39–6.28). Similarly, the risk of recurrence in patients with a Ki67 score of >20% prior to NST was more than fourfold compared with those with a Ki67 of ≤ 20% (HR 4.35; 95% CI 1.86–10.16). A reduction in treatment response by one Miller–Payne grade was associated with a 52% higher risk of recurrence (HR 1.52; 95% CI 1.16–2.00). There was a twofold higher risk of disease recurrence associated with an increase in cN status by one stage (HR 2.10; 95% CI 1.17–3.75).

**Fig. 2:**
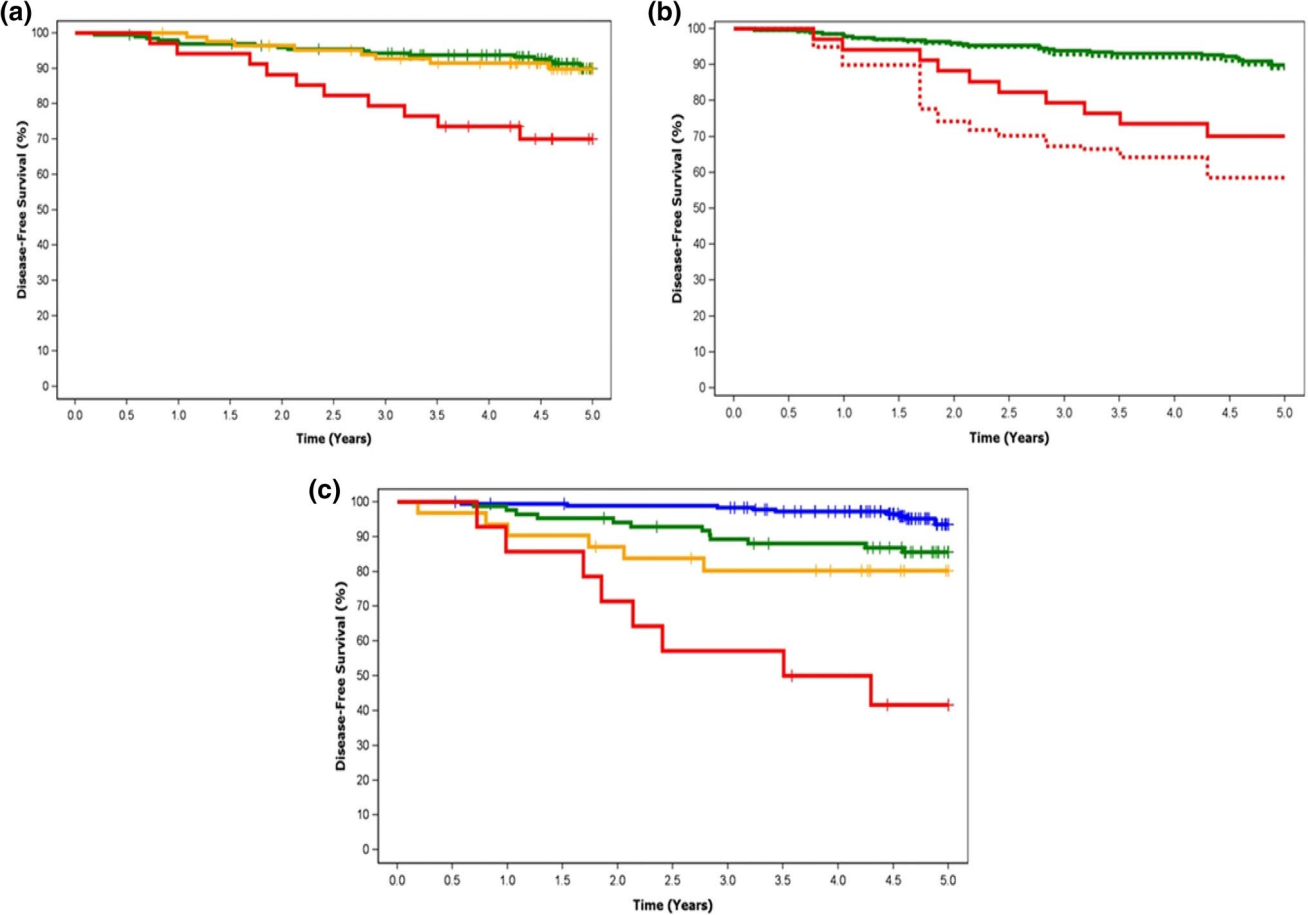
Kaplan–Meier estimates of 5-year disease-free survival, **a** stratified by TTL (TTL < 250 copies/µL [green], TTL 250–25,000 copies/µL [orange] and TTL > 25,000 copies/µL [red], **b** unadjusted (solid lines) or adjusted by Ki67 before NST, Miller–Payne grading and cN status (dotted lines) by TTL (TTL ≤ 25,000 copies/µL [green] and TTL > 25,000 copies/µL [red]), and **c** according to the DFS prognostic score, where Score 1 (blue) corresponds to a TTL of < 25,000 copies/µL, a Ki67 of ≤ 20% or Miller–Payne grade of 5, Score 2 (green) corresponds to TTL of ≥ 25,000 copies/µL, Ki67 > 20% or Miller–Payne grade of 3 or 4, Score 3 (orange) corresponds to a TTL of ≥ 25,000 copies/µL, Ki67 > 20% or Miller–Payne grade of 1 or 2, and Score 4 (red) corresponds to a TTL of ≥ 25,000 copies/µL and a Ki67 of > 20%. *DFS* disease-free survival, *NST* neoadjuvant systemic therapy, *TTL* total tumor load

There was no significant difference in DFS between patients with a TTL of < 250 copies/µL and those with a TTL of 250–25,000 copies/µL (Fig. 2a). However, DFS rates were significantly higher in patients with a TTL of ≤ 25,000 copies/µL compared with those patients with a TTL of > 25,000 copies/µL (89.9% vs. 70.0%, *p* = 0.0017; Fig. 2a). Adjustment to remove the effect of Ki67 before NST, Miller–Payne grading and cN status resulted in an even lower DFS for patients with a TTL of > 25,000 copies/µL (Fig. 2b).

A score to estimate 5-year DFS was derived, where DFS decreased with a high TTL (≥ 25,000 copies/µL), high Ki67 (> 20%), or low Miller–Payne grade (Grade 1 or 2; Fig. 2c).

## Discussion

Our study confirmed the predictive and prognostic value of TTL in breast cancer patients who had undergone surgery and SLNB after NST. TTL had stand-alone predictive value for non-SLN metastases (AUC = 0.87). A nomogram was constructed, based on TTL and clinicopathologic factors also identified as predictive of non-SLN involvement, and included a tumor size > 20 mm, and NST treatment response, and HER2-negative status in combination with a Ki67 ≤ 20%. TTL at a cut-off of 25,000 copies/µL was an independent prognostic factor for disease recurrence and DFS over 5-year follow-up period.

Previous studies showing that TTL determined from molecular analysis is an independent predictor of metastatic non-SLN in breast cancer patients differed markedly from ours by having excluded patients who had received NST and including only those with clinically negative ALNs [19, 23–25]. Only 62% of those in our study had clinically negative ALN after NST. Since our patients were treated with NST, they were more likely to have more aggressive or advanced disease than the patients in these prior studies. There were, however, some similarities with our patient population, including that all or most patients were cT1–3, most patients did not have lymphovascular infiltration, and all but one study [23] included a majority of patients with invasive ductal carcinoma.

The nomogram for prediction of non-SLN metastasis incorporated three parameters: TTL, tumor size, and a two-component histopathologic variable (HER2-negative tumor plus Ki67 of ≤ 20%). TTL and tumor size are parameters common to nomograms predicting non-SLN metastasis developed by other investigators [24–26]. For example, Shimazu and colleagues’ intraoperative nomogram included TTL (log TTL) and tumor size (non-binary) as predictive variables [25].

The amount of residual disease, especially in lymph nodes, after NST remains an important prognostic factor [4]. In our study, high TTL (> 25,000 copies/µL) after NST increased the risk of disease recurrence threefold compared with low TTL after NST. We found no significant difference in DFS prognosis between patients with a TTL of < 250 copies/µL versus ≥ 250–25,000 copies/µL, suggesting that small metastases (> 250 and < 25,000 copies/µL) detected by OSNA have similar prognostic value to negative nodes, i.e. their clinical outcome is no worse than a patient with negative nodes. A difference in DFS prognosis was found at a cut-off of 25,000 copies/µL, where DFS was significantly shorter among patients with TTL > 25,000 copies/µL than patients with TTL ≤ 25,000 copies/µL. We propose that this was related to prior administration of NST: a lower TTL would be expected in those in whom treatment was effective, indicating a greater likelihood of longer DFS (even if some macrometastases were found), whereas a high TTL after NST (> 25,0000 copies/µL) indicated a poor response to treatment and these patients would, therefore, be at greater risk of poor DFS. This difference in prognosis remained, even after adjusting for histopathologic grade, cN status and Ki67 score. Similar results for this TTL cut-off point have previously been reported in studies in patients that did not undergo prior NST [16, 17]. In one of these studies, TTL was prognostic of 5-year DFS at a cut-off of > 25,000 copies/µL (*p* = 0.041), and had been determined using intraoperative OSNA assay of SLNs [17].

We developed a prognostic scoring tool for DFS, which included TTL, the Ki67 score before NST, and treatment response in the primary tumor (Miller–Payne grade). This score includes TTL determined from molecular analysis of the SLN, and conventional prognostic markers, and could be considered as valuable as other scores of residual disease (for example, the Residual Cancer Burden [21]). The value of increased accuracy using molecular analysis of axillary node status has been previously demonstrated by many authors [6–12], and is further exemplified in our study by the observation that the proportion of patients with nodal involvement prior to NST was lower than that as determined after NST by OSNA assay (24.0% vs. 30.3%).

The potential clinical value of predicting non-SLN involvement using TTL and a nomogram among breast cancer patients post-NST is to spare the patient undergoing ALND when there is a low probability of ALN metastases. While the ACOSOG Z001 trial demonstrated that ALND can be avoided in a select group of SLN-positive patients [27], patients who have received NST such as those in our study do not meet ACOSOG Z001 criteria, and indeed application of these criteria seems to exclude the majority of SLN-positive patients [25]. Since an OSNA assay to determine TTL and thus non-SLN involvement can be performed intraoperatively [25, 26], it can also offer the advantage of clinical decision-making during primary surgery, avoiding a second surgery for ALND if required.

Study limitations attributable to the retrospective nature of the design included a lack of standardized NST and lack of a comparator control group. Also, molecular analysis by OSNA may not be as commonly available worldwide as it is in Europe and Japan. Finally, it may be argued by some that the use of whole nodes in the OSNA assay, precluding histologic analysis of the node, is a limitation. But multiple studies have shown that molecular assay is as accurate as conventional pathologic assessment, including in the post-NST setting [28].

In conclusion, this is the first fully published study to establish the predictive and prognostic value of TTL derived from molecular analysis of the SLN in breast cancer patients after NST. Non-SLN axillary involvement could be predicted using a nomogram. TTL also had prognostic value, allowing classification of patients into different risk grades of disease recurrence and probability of DFS. Although prospective studies are required to confirm our findings, our study supports the use of the intraoperative OSNA assay in breast cancer patients treated with NST.

## Supplementary Information

Below is the link to the electronic supplementary material.

Supplementary file1 (DOCX 25 KB)

Supplementary file2 (DOCX 32 KB)

Supplementary file3 (DOCX 52 KB)

## Data Availability

The datasets generated and/or analyzed during the current study are not publicly available due to confidentiality reasons but are available from the corresponding author on reasonable request.
