# Supplementary material for: Predictive and prognostic value of total tumor load in sentinel lymph nodes in breast cancer patients after neoadjuvant treatment using one-step nucleic acid amplification: the NEOVATTL study
Source: Clin Transl Oncol. 2021 Jan 31;23(7):1377–85. doi: 10.1007/s12094-020-02530-4 (PMC8192368; doi:10.1007/s12094-020-02530-4)
Supplement: Supplementary file 1 — Supplementary file1 (DOCX 25 KB) [file 12094_2020_2530_MOESM1_ESM.docx]

**Authors:**

Begoña Vieites^1^, María Ángeles López-García^1,11^, Maria Dolores Martín Salvago^2^, Cesar Luis Ramirez Tortosa^2^, Ricardo Rezola^3^, Sancho Magdalena^4^, Laura López Vilaró^5^, Felip Vilardell Villellas^6^, Octavio Burgués^7^, Beatriz Fernández-Rodriguez^8^, Lina Alfaro Galán^9^, Vicente Peg^10,11^

**Title:**

**“Predictive and prognostic value of total tumor load in sentinel lymph nodes in breast cancer patients after neoadjuvant treatment using one-step nucleic acid amplification: the NEOVATTL study”**

**Affiliations:**

^1^Department of Pathology - Hospital Universitario Virgen del Rocío (Sevilla, Spain)

^2^Department of Pathology - Hospital Universitario Materno-Infantil (Jaén, Spain)

^3^Department of Pathology - Onkologikoa Kutxa Fundazioa (Donostia, Spain)

^4^Department of Pathology - Hospital Universitario de Salamanca (Salamanca, Spain)

^5^Department of Pathology- Hospital de la Santa Creu i Sant Pau (Barcelona, Spain)

^6^Hospital Universitari Arnau de Vilanova (Lérida, Spain)

^7^Department of Pathology - Hospital Clínico Universitario de Valencia (Valencia, Spain)

^8^Department of Pathology - Complejo Hospitalario Universitario de Santiago (Santiago de Compostela, Spain)

^9^Department of Gynaecology and Obstetrics - Hospital Universitario Virgen del Rocío (Sevilla, Spain)

^10^Department of Pathology - Hospital Universitari Vall d'Hebron (Barcelona, Spain)

^11^CIBERONC (Centro de Investigación Biomédica en Red de Cáncer) – Instituto de Salud Carlos III (Madrid, Spain)

**Corresponding author:** Begoña Vieites

Email: [mb.vieites.sspa@juntadeandalucia.es](mailto:mb.vieites.sspa@juntadeandalucia.es)

# Online Resource 1

**Supplementary Methods**

Data collection

Data collected before neoadjuvant treatment: tumor stage, histologic subtype, estrogen receptor (ER) and progesterone receptor (PR) status, HER2 status, Ki67 proliferation index score, and sentinel lymph node biopsy (SLNB).

Neoadjuvant systemic therapy (NST) data collected: type of drugs and treatment scheme.

Data from breast and axilla surgery: type of surgery (conservative/radical), total tumor load (TTL), total number of removed SLN and non-SLN, the number of positive and negative SLN and non-SLN.

Data on pathologic tumor features after NST collected: tumor size, histologic grade and subtype, presence of lymphovascular invasion, hormone receptor status, HER2 status and Ki67, tumor stage, and response to treatment using Miller-Payne score.

Adjuvant therapy data collected: treatments (chemotherapy, hormonal treatments, and radiotherapy) and follow-up (outcome: alive, distant metastases, local relapse, regional relapse, recurrence).

Derivation of DFS prognostic score

To estimate 5-year disease-free survival, a prognostic scoring system was developed, as follows:

- If (TTL <25,000 copies/µL and Ki67 ≤20%) or (Miller-Payne grade = 5), then Score=1;
- If (TTL ≥25,000 copies/µL or Ki67 >20%) and (Miller-Payne grade = 3 or 4), then Score=2;
- If (TTL≥25,000 copies/µL or Ki67 >20%) and (Miller-Payne grade = 1 or 2), then Score=3;
- If TTL ≥ 25,000 copies/µL and Ki67 >20%, then Score=4.

If the patient meets the criteria for more than one score, then highest value score is chosen.
