# Supplementary material for: Predictive and prognostic value of total tumor load in sentinel lymph nodes in breast cancer patients after neoadjuvant treatment using one-step nucleic acid amplification: the NEOVATTL study
Source: Clin Transl Oncol. 2021 Jan 31;23(7):1377–85. doi: 10.1007/s12094-020-02530-4 (PMC8192368; doi:10.1007/s12094-020-02530-4)
Supplement: Supplementary file 2 — Supplementary file2 (DOCX 32 KB) [file 12094_2020_2530_MOESM2_ESM.docx]

# Online Resource 2

**Authors:**

Begoña Vieites^1^, María Ángeles López-García^1,11^, Maria Dolores Martín Salvago^2^, Cesar Luis Ramirez Tortosa^2^, Ricardo Rezola^3^, Sancho Magdalena^4^, Laura López Vilaró^5^, Felip Vilardell Villellas^6^, Octavio Burgués^7^, Beatriz Fernández-Rodriguez^8^, Lina Alfaro Galán^9^, Vicente Peg^10,11^

**Title:**

**“Predictive and prognostic value of total tumor load in sentinel lymph nodes in breast cancer patients after neoadjuvant treatment using one-step nucleic acid amplification: the NEOVATTL study”**

**Affiliations:**

^1^Department of Pathology - Hospital Universitario Virgen del Rocío (Sevilla, Spain)

^2^Department of Pathology - Hospital Universitario Materno-Infantil (Jaén, Spain)

^3^Department of Pathology - Onkologikoa Kutxa Fundazioa (Donostia, Spain)

^4^Department of Pathology - Hospital Universitario de Salamanca (Salamanca, Spain)

^5^Department of Pathology- Hospital de la Santa Creu i Sant Pau (Barcelona, Spain)

^6^Hospital Universitari Arnau de Vilanova (Lérida, Spain)

^7^Department of Pathology - Hospital Clínico Universitario de Valencia (Valencia, Spain)

^8^Department of Pathology - Complejo Hospitalario Universitario de Santiago (Santiago de Compostela, Spain)

^9^Department of Gynaecology and Obstetrics - Hospital Universitario Virgen del Rocío (Sevilla, Spain)

^10^Department of Pathology - Hospital Universitari Vall d'Hebron (Barcelona, Spain)

^11^CIBERONC (Centro de Investigación Biomédica en Red de Cáncer) – Instituto de Salud Carlos III (Madrid, Spain)

**Corresponding author:** Begoña Vieites

Email: [mb.vieites.sspa@juntadeandalucia.es](mailto:mb.vieites.sspa@juntadeandalucia.es)

**Supplementary Table S1** Pathologic tumor features after NST, stratified by total tumor load

|  | **Total tumor load (copies/µL)** | | | | | |  | |
| --- | --- | --- | --- | --- | --- | --- | --- | --- |
|  | **<250** | | **250–25,000** | | **>25,000** | | **All patients** | |
|  | **Before NST** | **After NST** | **Before NST** | **After NST** | **Before NST** | **After NST** | **Before NST** | **After NST** |
| Maximum tumor size | *n*=167 | *n*=192 | *n*=67 | *n*=83 | *n*=33 | *n*=34 | *n*=267 | *n*=309 |
| pCR | - | 69 (35.9) | NA | NA | NA | NA | - | 69 (22.3) |
| 0 mm | - | - | - | 11 (13.3) | - | 1 (2.9) | - | 12 (3.9) |
| ≤20 mm | 13 (7.8) | 75 (39.1) | 8 (11.9) | 38 (45.8) | 4 (12.1) | 12 (35.3) | 25 (9.4) | 125 (40.5) |
| >20 mm | 154 (92.2) | 48 (25.0) | 59 (88.1) | 34 (41.0) | 29 (87.9) | 21 (61.8) | 242 (90.6) | 103 (33.3) |
| Tumor histologic subtype | *n*=196 | *n*=194 | *n*=83 | *n*=73 | *n*=33 | *n*=33 | *n*=312 | *n*=300 |
| pCR | NA | 69 (35.6) | NA | NA | NA | NA | NA | 69 (23.0) |
| Invasive NOS | 167 (85.2) | 95 (49.0) | 70 (84.3) | 60 (82.2) | 25 (75.8) | 27 (81.8) | 262 (84.0) | 182 (60.7) |
| Others | 29 (14.8) | 30 (15.5) | 13 (15.7) | 13 (17.8) | 8 (24.2) | 6 (18.2) | 50 (16.0) | 49 (16.3) |
| Histologic grade | *n*=187 | *n*=191 | *n*=79 | *n*=71 | *n*=30 | *n*=33 | *n*=296 | *n*=295 |
| pCR | NA | 69 (36.1) | NA | NA | NA | NA | NA | 69 (23.4) |
| G1 | 17 (9.1) | 29 (15.2) | 11 (13.9) | 22 (31.0) | 8 (26.7) | 11 (33.3) | 36 (12.2) | 62 (21.0) |
| G2 and G3 | 170 (90.9) | 93 (48.7) | 68 (86.1%) | 49 (69.0) | 22 (73.3) | 22 (66.7) | 260 (87.8) | 164 (55.6) |
| *In situ* carcinoma | *n*=196 | *n*=167 | *n*=84 | *n*=69 | *n*=34 | *n*=25 | *n*=314 | *n*=261 |
| No | 157 (80.1) | 108 (64.7) | 65 (77.4) | 38 (55.1) | 28 (82.4) | 16 (64.0) | 250 (79.6) | 162 (62.1) |
| Yes | 39 (19.9) | 59 (35.3) | 19 (22.6) | 31 (44.9) | 6 (17.6) | 9 (36.0) | 64 (20.4) | 99 (37.9) |
| LVI | *n*=196 | *n*=174 | *n*=84 | *n*=79 | *n*=34 | *n*=33 | *n*=314 | *n*=286 |
| No | 126 (64.3) | 133 (76.4) | 51 (60.7) | 45 (57.0) | 22 (64.7) | 14 (42.4) | 199 (63.4) | 192 (67.1) |
| Yes | 70 (35.7) | 41 (23.6) | 33 (39.3) | 34 (43.0) | 12 (35.3) | 19 (57.6) | 115 (36.6) | 94 (32.9) |
| ER | *n*=196 | *n*=67 | *n*=82 | *n*=33 | *n*=33 | *n*=11 | *n*=311 | *n*=111 |
| Negative | 71 (36.2) | 19 (28.4) | 10 (12.2) | 1 (3.0) | 3 (9.1) | 1 (9.1) | 84 (27.0) | 21 (18.9) |
| Positive (≥1%) | 125 (63.8) | 48 (71.6) | 72 (87.8) | 32 (97.0) | 30 (90.9) | 10 (90.9) | 227 (73.0) | 90 (81.1) |
| PR | *n*=194 | *n*=67 | *n*=81 | *n*=33 | *n*=30 | *n*=11 | *n*=305 | *n*=111 |
| Negative | 93 (47.9) | 34 (50.7) | 17 (21.0) | 18 (54.5) | 6 (20.0) | 3 (27.3) | 116 (38.0) | 55 (49.5) |
| Positive (≥1%) | 101 (52.1) | 33 (49.3) | 64 (79.0) | 15 (45.5) | 24 (80.0) | 8 (72.7) | 189 (62.0) | 56 (50.5) |
| HER2 | *n*=196 | *n*=182 | *n*=84 | *n*=80 | *n*=34 | *n*=31 | *n*=314 | *n*=293 |
| Negative | 135 (68.9) | 175 (96.2) | 70 (83.3) | 73 (91.3) | 28 (82.4) | 30 (96.8) | 233 (74.2) | 278 (94.9) |
| Positive | 61 (31.1) | 7 (3.8) | 14 (16.7) | 7 (8.8) | 6 (17.6) | 1 (3.2) | 81 (25.8) | 15 (5.1) |
| Ki67 (%) | *n*=194 | *n*=64 | *n*=83 | *n*=33 | *n*=33 | *n*=10 | *n*=310 | *n*=107 |
| ≤20% | 69 (35.6) | 40 (62.5) | 38 (45.8) | 24 (72.7) | 19 (57.6) | 8 (80.0) | 126 (40.6) | 72 (67.3) |
| >20% | 125 (64.4) | 24 (37.5) | 45 (54.2) | 9 (27.3) | 14 (42.4) | 2 (20.0) | 184 (59.4) | 35 (32.7) |
| Data are *n* (%)  ER, estrogen receptor; LVI, lymphovascular infiltration; NA, not applicable; NST, neoadjuvant systemic therapy; pCR, pathologic complete response; PR, progesterone receptors; TTL, total tumor load | | | | | | | | |
