# Supplementary material for: Predictive and prognostic value of total tumor load in sentinel lymph nodes in breast cancer patients after neoadjuvant treatment using one-step nucleic acid amplification: the NEOVATTL study
Source: Clin Transl Oncol. 2021 Jan 31;23(7):1377–85. doi: 10.1007/s12094-020-02530-4 (PMC8192368; doi:10.1007/s12094-020-02530-4)

**Authors:**

Begoña Vieites^1^, María Ángeles López-García^1,11^, Maria Dolores Martín Salvago^2^, Cesar Luis Ramirez Tortosa^2^, Ricardo Rezola^3^, Sancho Magdalena^4^, Laura López Vilaró^5^, Felip Vilardell Villellas^6^, Octavio Burgués^7^, Beatriz Fernández-Rodriguez^8^, Lina Alfaro Galán^9^, Vicente Peg^10,11^

**Title:**

**“Predictive and prognostic value of total tumor load in sentinel lymph nodes in breast cancer patients after neoadjuvant treatment using one-step nucleic acid amplification: the NEOVATTL study”**

**Affiliations:**

^1^Department of Pathology - Hospital Universitario Virgen del Rocío (Sevilla, Spain)

^2^Department of Pathology - Hospital Universitario Materno-Infantil (Jaén, Spain)

^3^Department of Pathology - Onkologikoa Kutxa Fundazioa (Donostia, Spain)

^4^Department of Pathology - Hospital Universitario de Salamanca (Salamanca, Spain)

^5^Department of Pathology- Hospital de la Santa Creu i Sant Pau (Barcelona, Spain)

^6^Hospital Universitari Arnau de Vilanova (Lérida, Spain)

^7^Department of Pathology - Hospital Clínico Universitario de Valencia (Valencia, Spain)

^8^Department of Pathology - Complejo Hospitalario Universitario de Santiago (Santiago de Compostela, Spain)

^9^Department of Gynaecology and Obstetrics - Hospital Universitario Virgen del Rocío (Sevilla, Spain)

^10^Department of Pathology - Hospital Universitari Vall d'Hebron (Barcelona, Spain)

^11^CIBERONC (Centro de Investigación Biomédica en Red de Cáncer) – Instituto de Salud Carlos III (Madrid, Spain)

**Corresponding author:** Begoña Vieites

Email: [mb.vieites.sspa@juntadeandalucia.es](mailto:mb.vieites.sspa@juntadeandalucia.es)

# Online Resource 3

**Supplementary Fig. S1** ROC curve based on the sensitivity and the specificity of TTL for the prediction of non-SNL involvement. ROC, receiver operator characteristic; SNL, sentinel lymph node; TTL, total tumor load


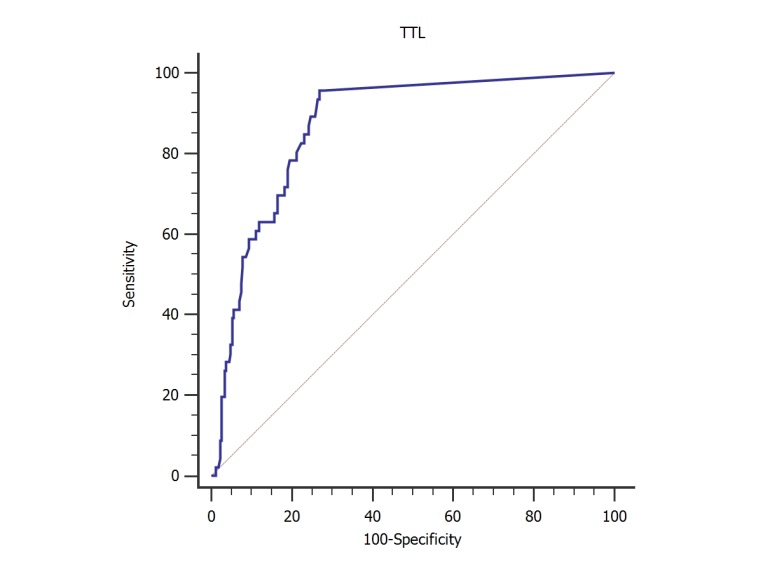

Supplement: Supplementary file 3 — Supplementary file3 (DOCX 52 KB) [file 12094_2020_2530_MOESM3_ESM.docx]
